# A case report of a self-inserted foreign body in the urethra/bladder causing urinary calculus formation, and a review of the literature

**DOI:** 10.1016/j.heliyon.2023.e14038

**Published:** 2023-02-26

**Authors:** Amirreza Fotovat, Samira Yavari, Mohsen Ayati, Mohammad Reza Nowroozi, Laleh Sharifi

**Affiliations:** a Uro-Oncology Research Center, Tehran University of Medical Sciences, Tehran, Iran; b Department of Anesthesiology, School of Medicine, Esfahan University of Medical Sciences, Esfahan, Iran

**Keywords:** Lower genitourinary system, Foreign body, Bladder, Urethra, Hydronephrosis, Bladder stone

## Abstract

Several self-inserted foreign bodies have been reported in the lower genitourinary system. We report a 27-year-old man with suprapubic severe pain, purulent discharge from the urethra**,** and dribbling. He had a history of psychotic disorders and inserting an ink chamber of a pen into the urethra. Imaging showed hydronephrosis and a large urinary stone in the bladder with no sign of foreign body. During open cystotomy, we found that bladder stone was attached to a plastic tube that was extended into the patient's urethra. In such cases, timely surgery to prevent urinary retention and psychological support are required.

## 1 Introduction

Several self-inserted foreign bodies have been reported in the lower genitourinary system, such as screws, pens, nuts, cables, etc. [1].

Foreign objects in the lower urinary tract are often associated with long-term complications such as urinary tract infection (UTI), hematuria, increased urinary frequency, dysuria, urethral false passages, stricture, fistula, and pain [2].

Self-insertion of objects into the urinary tract is quite rare in emergency visits. Imaging is necessary to detect the position, shape, and size of foreign bodies [1].

Here, we report a male patient with transurethral self-insertion of the ink chamber of a pen into the urethra which caused a large calculus formation in the bladder.

**Fig. 1:**
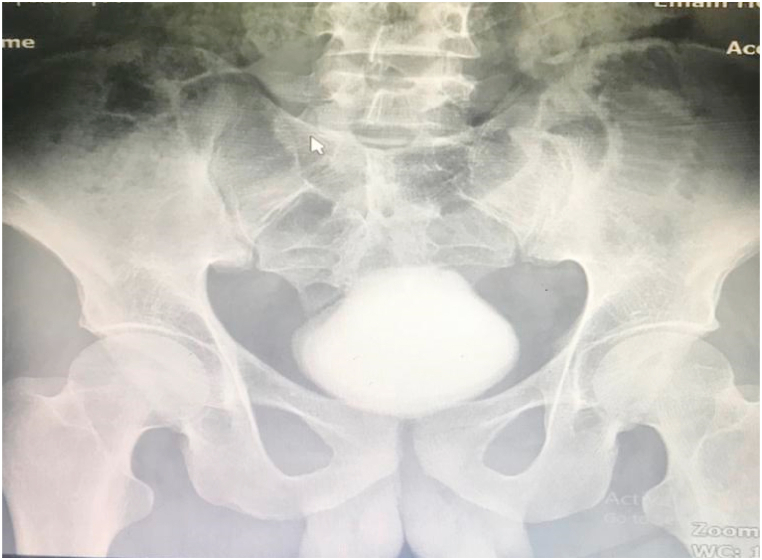
Abdomen X-ray of the patient, showed a large urinary calculus in the bladder.

**Fig. 2:**
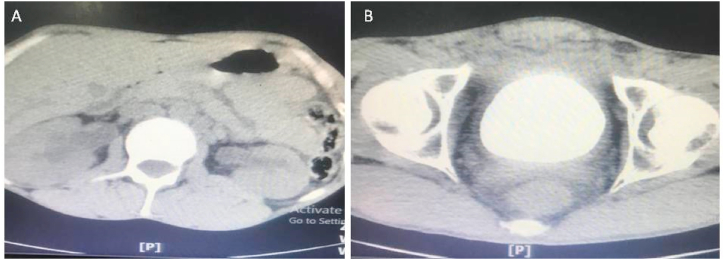
CT scan without contrast showed: A-mild to moderate bilateral hydronephrosis, B- a large urinary calculus in the bladder.

Case presentation:

A 27-year-old man with the chief complaint of suprapubic severe pain, purulent discharge from the urethra**,** and dribbling was referred to a tertiary private clinic in Golpaygan, Iran. He declared a history of psychotic disorders and initiation of the signs after he attempted to insert a pen tube into the urethra. Laboratory examination confirmed a severe urinary tract infection (UTI) and creatinine equal to 2. X-ray and CT scan showed a large urinary stone in the bladder occupying almost all of the intravesical space with no sign of foreign object (Fig. 1, Fig. 2B), and mild to moderate bilateral hydronephrosis (Fig. 2A). The patient underwent open cystotomy with an initial diagnosis of bladder calculus and the possibility of a foreign body in the urethra and bladder.

**Fig. 3:**
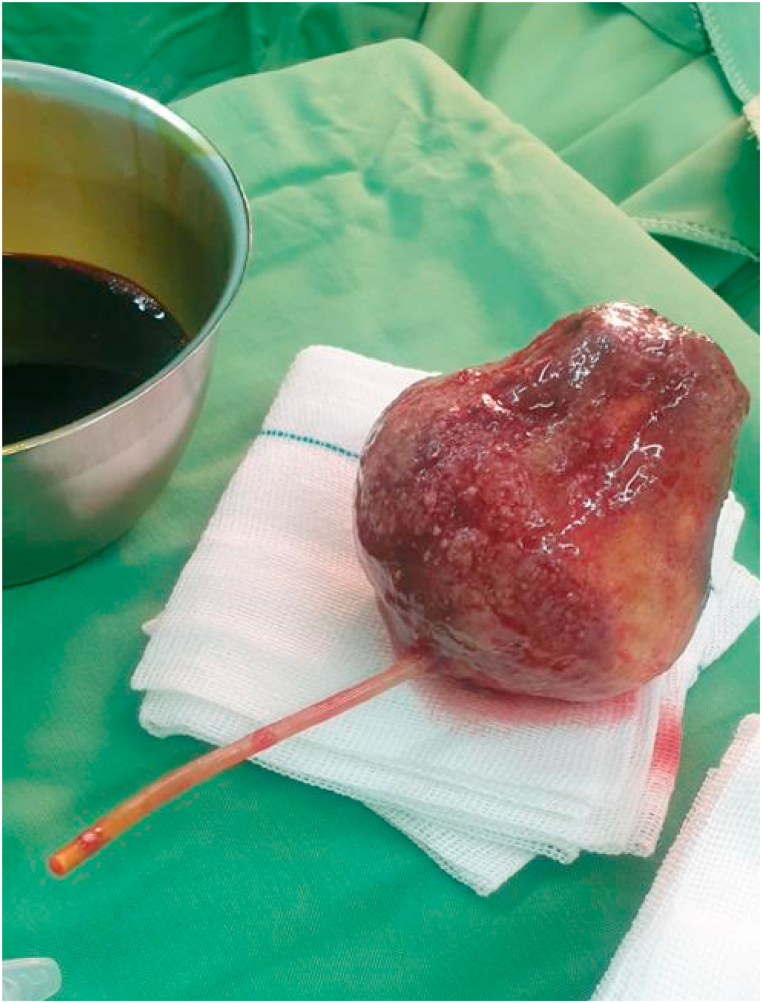
Removed bladder calculus attached to plastic ink chamber of pen.

After opening the bladder during the surgical procedure, we discovered that the bladder stone was attached to a plastic tube that was extended into the patient's urethra (Fig. 3). According to the patient's history, we found out that the patient tried to insert a pen ink chamber inside the urethra, and probably it's plastic material and ink secretions led to bladder irritation and stone formation.

Two days later, the patient was discharged in good general condition. The patient followed for checking the recovery status after surgery after 3 months. He had no pain or any sign of urethral obstruction and urinary retention. Blood examinations including UA, UC, and creatinine were normal. Control sonography showed no sign of hydronephrosis or complications such as a urethral stricture. The patient was referred to a psychology clinic to prevent other probable problems.

Written informed consent was obtained from the patient for the publication of the collection of his clinical data.

## 2 Discussion

Mostly, foreign bodies have been reported in the respiratory and gastrointestinal systems, but there is limited literature on the case of the lower urinary system. including case reports and small case series and there is a lack of data regarding management and epidemiology in this regard [3]. Some of the unusual foreign bodies have been reported to be self-inserted into the urethral/vesicle such as olive seeds [4], Allen key [5], Tongue cleaner [6], Nail scissor [7], a telephone cable [8].

Dayron Rodríguez et al., in 2020 showed that genitourinary foreign bodies have a crucial burden on healthcare systems. They found that they occurred frequently in younger female patients but penile and urethral/bladder foreign objects are more frequent in older men and were related to longer hospital admission days and costs [3].

Some behaviors such as autoerotic stimulation, intoxication, senility, Psychosis With or Without Mood Disturbance, Cognitive Disorders, Malingering, and stash of drug through the urethra into the bladder to escape prosecution are reported as the cause of the self-insertion of foreign objects [[9], [10], [11]]. Even, a suicidal attempt through the transurethral insertion of a cylindrical foreign body via the urethra causing bladder perforation has been reported. Autoerotic or accidental death was ruled out by psychological autopsy and this unique and painful method of suicide was confirmed [12].

Almost one-third of male patients who suffered urethra/bladder foreign bodies had a history of major mental disorders [3]. In addition to cistolithotomy and stone removal, patients should undergo a timely psychological evaluation. Determining predisposing factors of foreign body insertion can lead to management approaches that affect behavior motivation. Failure to recognize the underlying cause leaves the patient at risk of repeated injury. Medical personnel should be qualified to come across such patients. Sometimes, staff reactions such as confusion, laughter, derision, fear, embarrassment, and anger to such behaviors are severe and can disturb their compassionate role in treatment [11].

Most foreign objects in the urinary system can be removed endoscopically but in the case of our patient, open surgical removal was necessary due to the large calculi formation in the bladder.

Urethral stenosis is common after the removal of foreign objects because of tissue injury; therefore, antibiotics and tetanus prophylaxis are essential after surgical interventions [1].

Foreign bodies in the bladder are the main risk factor for larger bladder stones. Any foreign body that makes its way into the bladder can act as scaffolding for calculus construction. The formation of stones is a step-by-step mechanism that has been well presented in the literature [13]. Also, there are reports that urinary calculus in the bladder is associated with the risk of renal failure [14].

Three reasons are described for the miss-diagnosis of self-inserted urethral foreign bodies. The first reason is the rare occurrence of this situation. Second is not having a correct history of the disease due to the unwillingness of patients to give information and third is the inefficiency of simple X-ray imaging to detect radiolucent objects [2]. Although, our patient met all the possibilities of miss-diagnosis, fortunately, he underwent tempestive treatment due to the formation of a large stone in the bladder. Our case had developed bilateral hydronephrosis and mild renal failure because of his large bladder stone; but, timely surgery prevented bladder outlet obstruction and medical examination 3 months after the operation confirmed his complete recovery.

## 3 Conclusion

The presented patient in this report was a case of self-inserted *trans*-urethral ink chamber of a pen leading to a large stone formation in his bladder. Timely removal of the stone prevented bladder outlet obstruction and reversed hydronephrosis. Psychological counseling and psychotherapy can be effective in preventing the recurrence of such cases.

## Author contribution statement

All authors listed have significantly contributed to the investigation, development and writing of this article. </p>

## Funding statement

This research did not receive any specific grant from funding agencies in the public, commercial, or not-for-profit sectors.

## Data availability statement

No data was used for the research described in the article.

## Additional information

No additional information is available for this paper.

## Declaration of competing interest

The authors declare no competing interests.
